# Antiphospholipid antibodies are associated with increased levels of selected oxidative stress biomarkers

**DOI:** 10.1186/s12959-025-00762-4

**Published:** 2025-07-30

**Authors:** Weronika Nowak, Joanna Kołodziejczyk-Czepas, Oleksandra Liudvytska, Marzena Tybura-Sawicka, Emilia Krzemińska, Anna Puła, Jacek Treliński

**Affiliations:** 1 Department of Hemostasis Disorders, Medical University of Lodz, Pabianicka 62, Łódź, 93-513 Poland; 2 Department of General Biochemistry, Faculty of Biology and Environmental Protection, University of Lodz, Pomorska 141/143, Lodz, 90-236 Poland; 3 Department of Hematology, Copernicus Memorial Hospital in Lodz, Pabianicka 62, Łódź, Poland; 4 Department of Hematooncology, Copernicus Memorial Hospital in Lodz, Pabianicka 62, Łódź, Poland

**Keywords:** Antiphospholipid antibodies, Oxidative stress biomarkers, Nitrative stress biomarkers, Thrombosis, ROTEM

## Abstract

**Background:**

Antiphospholipid antibodies (aPLs) are detected in 1–5% of the general population. They include lupus anticoagulant (LAC), anticardiolipin antibodies (aCL) and anti-β2-glycoprotein I antibodies (aβ2GPI). APL increases thrombotic risk, but the pathogenesis of this effect is not fully understood.

**Objectives:**

The aim of this study was to evaluate oxidative and nitrosative stress biomarkers and their relation to certain rotational thromboelastometry (ROTEM) parameters as a risk factor for thrombosis in 32 patients in whom the presence of antiphospholipid antibodies was confirmed, but who had never experienced a thrombosis event (Group 1) in order to rule out any impact of thrombosis on stress parameters. The parameters were also assessed in a group of 23 healthy volunteers (Group 2).

**Methods:**

To assess FRAP and thiol groups we used colorimetric method. The level of protein carbonylation, total pool of 3-nitrotyrosine in plasma proteins, 3-nitrotyrosine-containing fibrinogen as well as the acetyl-lysine-containing fibrinogen were estimated by ELISA. Lipid hydroperoxides were detected using the ferric-xylenol orange hydroperoxide assay. Additionally four ROTEM tests, i.e. INTEM, EXTEM, FIBTEM and APTEM, were performed. In statistical analysis the Mann-Whitney U-test, Student’s t-test and logistic regression were used.

**Results:**

TBARS (*p* = 0,002), LOOH (*p* = 0,035) and carbonyl groups (*p* = 0,018) were markedly higher in Group 1 compared to Group 2. Also the acetyl-lysine-containing fibrinogen were significantly higher in Group 1 (*p* = 0,0028). Other biomarkers did not differ markedly between the studied groups. The obtained results of ROTEM, were not consistent and did not clearly indicate hypercoagulable state.

**Conclusion:**

Study confirms increased levels of oxidative biomarkers in patients in whom the presence of antiphospholipid antibodies was confirmed, but who had never experienced a thrombosis event. Oxidative stress may an important role in the pathogenesis of APS and is not secondary to thrombosis.

## Introduction

Antiphospholipid antibodies (aPLs) are detected in 1–5% of the general population [1]. They include lupus anticoagulant (LAC), anticardiolipin antibodies (aCL) and anti-β2-glycoprotein I antibodies (aβ2GPI). For the diagnosis of antiphospholipid syndrome there must be presence aPLs and arterial and/or venous thrombosis and/or pregnancy morbidity. APS has heterogeneous clinical manifestation and can also be accompanied by other morbid features like thrombocytopenia, accelerated atherosclerosis, cardiac dysfunction or cognitive decline [2]. APS is also a leading cause of strokes in people under 50 years old [3]. 

The pathogenesis of APS is not fully understood. One of the hypotheses suggests that circulating aPLs destroy the integrity of the endothelium and induce the procoagulant phenotype, yet that is not enough to cause thrombosis [4]. Another triggering factor is needed to provoke thrombosis, such as for example acute infections, oxidative stress or inflammation. Recent studies demonstrate the key role of oxidative stress (OS) in thrombosis [5]. OS is an imbalance between the production and accumulation of oxygen reactive species (ROS) in cells and tissues and the ability of the biological system to detoxify these radical and non-radical products. Overproduction of ROS leads to DNA damage, lipid peroxidation and oxidative protein modifications [6]. The involvement of ROS in protein post-translational modifications (PTMs) is complemented by indirect changes such as acetylation, glycosylation, phosphorylation and citrullination [7]. Such modifications cause conformational changes in proteins and affect their function [2]. 

Despite the involvement of oxidative stress in pathology of many diseases being well-described [8, 9] disturbances in antioxidant-pro-oxidant balance at different stages of APS development are still only fragmentarily recognized. However, there is some in vitro and in vivo evidence indicating a link between disorders appearing in APS and oxidative stress. For instance, it has been shown that aPLs can disrupt antioxidant-pro-oxidant balance, including stimulation of the superoxide dismutase (SOD) expression in leukocytes, mitochondrial overload, enhancement in ROS generation, and induction of the pro-oxidative state [10, 11]. There are several other works indicating the involvement of oxidative stress in the pathogenesis of APS, including the mechanisms promoting OS and mechanisms mediated by OS contributing to thrombotic complications [2, 4, 12]. Ames [13] were first to published a study looking at oxidative stress in primary antiphospholipid syndrome (PAPS), in which the levels of F2-isoprostanes were significantly higher in PAPS than in healthy control. Increased levels of 8-isoprostanes were also reported in independent studies, by Ferro [14] and Sciascia [15]. Matsuura [16] reported increased levels of oxidized low-density lipoproteins (oxLDL), while Nojima [12] noted an increased oxidative stress index. Moreover Stanisavljevic [17], Lambert [18] and Delgado [19], in independent studies, described reduced paraoxonase 1 activity (PON1). PON1 is a hydrolytic enzyme which prevents lipid oxidation. On the other hand, Vaz et al. [20] found no differences between the levels of malondialdehyde (MDA), carbonylated proteins, and 8-isoprostanes in plasma from APS patients versus healthy volunteers.

In addition to the overproduction of oxidants such as superoxide anion or other oxygen radicals, disruption of the antioxidant-pro-oxidant balance may be enhanced by the nitric oxide synthase (iNOS)-mediated nitrative and nitrosative stress. The nitrative stress, a results of an excessive generation of nitrogen oxide and other reactive nitrogen species (peroxynitrite, in particular) contributes to disorders occurring at different molecular and physiological levels, such as changes in signaling pathways, enzyme inhibition and alterations in cell functionality. The expression and activity of the iNOS enzyme are strictly associated with a triggering inflammatory and immune response. Overexpression or disregulation of iNOS has been implicated in numerous pathologies, including immune diseases [21]. Furthermore, due to its ability to continuously generate large amounts of nitric oxide, the iNOS enzyme is one of the factors that are critical for the generation of peroxynitrite and for promotion of the nitrative stress [22]. 

The majority of OS studies involved patients with APS after a thrombotic event, hence it was impossible to assess if the oxidative stress was a pathogenetic factor or was increased due to sustained thrombosis. We decided to assess different oxidative stress markers and to related them with ROTEM parameters in a group of participants in whom the presence of antiphospholipid antibodies was confirmed, but who had never experienced thrombosis event, in order to rule out any impact of thrombosis on stress parameters. ROTEM is a sensitive marker of coagulation disorders, which also can be useful in the evaluation of hypercoagulation state (reduction in CFT, increase in MCF, higher alpha angle). We assessed a broad panel of oxidative stress biomarkers, including the ferric-reducing ability of plasma (FRAP), nitric oxide (NO) level as well as markers related to plasma proteins (thiol groups, carbonyl groups) and to lipids (TBARS, LOOH), which provide data on the efficiency of different pathways of biomolecule oxidation in the APS patients. Taking into consideration the data on the role of nitrative and nitrosative stress in pathologies related to the cardiovascular system [23], multiple myeloma [24] and immune diseases [25], we included the aspect of nitrative stress biomarkers into our study as well. As nitrated fibrinogen was found to be elevated in the setting of acute VTE, we focused on effects of nitrative stress in the context of this protein.

## Materials and methods

The study included two groups: Group 1 consisting of 32 participants with confirmed antiphospholipid antibodies without previous thromboembolic event and Group 2 consisting of 23 healthy volunteers. We identified a group of patients with aPLs when diagnosing patients with prolonged APTT and women with pregnancy loss. The aPLs were tested twice and for ACA and B2-GPI we only picked out patients with a high titer of antibodies (> 40 GPL).

The study protocol was approved by the local ethics committee (Medial University of Łódź, No. RNN/196/21/KE- 13.07.2021). All participants provided informed consent.

The exclusion criteria were as follows: known liver disorder (plasma alanine transaminase concentration > 2 upper limit range), renal failure (creatinine concentration ≥ 2 mg/dL), thrombocytopenia (platelet count < 100 × 10⁹ /l), previous thromboembolic event and taking any drug that strongly influences platelet function or coagulation for 10 days prior to study entry. The characteristics of the study population are shown shown in Table 1. The groups did not differ significantly in terms of age, leukocyte level, hemoglobin level, platelet count, fibrinogen level and hypercoagulability markers (e.g. deficiency of protein C, antithrombin level, factor VIII activity).

**Table 1:** Characteristics of study population

Parameters	GROUP 1	GROUP 2	*p*
Median age in years (range)	67.5 (27–82)	56.7 (30–57)	ns
Sex (F/M)	28/4	20/3	
WBC	5.7 (3.08–11.22)	6.2 (3.3–10.7)	ns
Hb	14 (10.3–15.6)	14 (12.5–16.6)	ns
PLT	228 (122–506)	258 (163–330)	ns
APTT	62.2 (27.5-138.6)	26.6 (22.9–32.3)	< 0.001
PT	12 (10.5–15.8)	11.4 (10.4–13)	0.023
Fibrinogen	318 (205–670)	301 (215–597)	ns
Fibrinogen-nefelometric method	3.45 (2.4–6.3)	3.1 (2.4–5.8)	ns
VIII	101 (30–162)	114 (104–124)	ns
Antithrombin III	106 (82–119)	95.5 (78–124)	ns
Protein C	111 (102–140)	119 (110–145)	ns
Protein S	114 (75.8–135)	133 (75.6-149.9)	ns
CRP	1.95 (0.6–23.9)	2.6 (0.6–14.1)	ns
IL-6	5.2 (1.7–20.8)	3.1 (1.6–6.6)	ns
TNF	26.2 (6-67.8)	15.3 (8.7–62.0)	0.047

ns - not significant; F - female; M - male; PLT – platelets G/L; PT - prothrombin time (s); APTT - activated partial thromboplastin time (s), CRP- C-reactive protein (mg/l), Hb - hemoglobin level (g/dl), WBC - white blood cells (G/L), IL-6-interleukin (pg/ml); TNF- tumor necrosis factor (pg/ml)

p-statistical significance, ns- not significant

### Chemicals

The phosphate-buffered saline (PBS), tris(hydroxymethyl)aminomethane, 5,5′-dithio-bis(2-nitrobenzoic) acid (DTNB), thiobarbituric acid, trichloroacetic acid, sulphuric acid, SIGMAFAST™ OPD (*o*-phenylenediamine dihydrochloride) chromogenic substrate, rabbit anti-dinitrophenyl (anti-DNP antibody, #D9656), secondary anti-rabbit antibody conjugated with peroxidase (#A0545), and the peroxidase-conjugated anti-fibrinogen antibody (#A9452) were purchased from Aldrich (Sigma-Aldrich, St. Louis, USA). 2,4,6-Tris(2-pyridyl)-s-triazine (TPTZ) and xylenol orange were purchased from Merck (Darmstadt, Germany). Other antibodies for the enzyme-linked immunosorbent assay (ELISA)-based detections, i.e., anti-acetyl lysine antibody (#ab80178), primary anti-3-nitrotyrosine antibody (#ab20117), and Streptavidin/ HRP complex (#ab7003) were purchased from Abcam (Cambridge, UK). Biotinylated secondary antibody (#31732) for ELISA (3-NT detection) and Pierce™ BCA Protein Assay Kit (#23227) was purchased from ThermoFisher Scientific (Waltham, MA, USA). All other organic and inorganic reagents (of analytical grade) were purchased from Alfachem (Lublin, Poland).

### Detection of oxidative stress biomarkers in blood plasma

#### Measurements of the ferric-reducing ability of plasma (the FRAP assay)

The FRAP assay was used to estimate the non-enzymatic antioxidant capacity of blood plasma, based on the reducing activity of low-molecular antioxidants that are present in blood plasma and which reduce ferric (Fe^3+^) to ferrous (F^2+^) ions. The assay was a modified colorimetric method of Benzie and Strain [26]. Briefly, plasma was diluted 4 times with 0.9% NaCl, transferred into microplate wells (20 µl/well). Then, the working reagent (composed of 300 mM acetate buffer (pH 3.6), 10 mM TPTZ (in 0.04 M HCl), and 20 mM FeCl_3_) was added. The volume ratio for diluted plasma, acetate buffer, TPTZ and FeCl_3_ was of 1:10:1:1, respectively. After 15-min incubation at 37 °C, absorbance was measured at λ = 593 nm. Results were calculated from the standard curve (prepared from FeSO_4_ solutions) and expressed as equivalents of Fe^2+^.

#### Carbonyl groups

The level of protein carbonylation was estimated by ELISA, based on a modified method of Alamdari et al. [27] Briefly, plasma samples were diluted with 0.02 M phosphate-buffered saline, to obtain a final concentration of 5 µg of protein/ml, and layered (200 µl) into polystyrene microplate wells. Following an overnight incubation at 4 °C, microplates were washed three times with 0.02 M PBS (250 µl/well). Then, samples were incubated in the dark with the DNPH solution (0.05 mM, pH 6.2; 200 µl/well, for 45 min). For complete removal of the unreacted DNPH, a two-step washing procedure was applied: firstly, each well was washed five times with the 250 µl of 0.02 M PBS/ethanol mixture (1:1; v/v); next, the well was washed with one 250 µl volume of 0.02 M PBS. Then, the well surface was blocked with defatted milk solution (5%, in 0.02 M PBS; 250 µl/well) for 1.5 h, at 37 °C. The milk solution was removed, and microplate wells were washed three times with 0.02 M PBS, enriched with 0.01% Tween 20 (PBST; 250 µl/well). Then, the anti-DNP antibody solution (1:2500) was added (200 µl/well), and the samples were incubated at 37 °C, for 1 h. Unbound anti-DNP antibody was removed by washing 5-times with PBST (250 µl/well). Then, the horseradish peroxidase-conjugated secondary antibody solution (1:2500) was added (200 µl/well). After 1 h of incubation at 37 °C, unbound secondary antibodies were removed by washing 5-times with PBST (250 µl/well). For the visualization, the SIGMAFAST™ OPD (*o*-phenylenediamine dihydrochloride) substrate solution (200 µl/well) was added. The reaction was stopped with 50 µl of 40% sulphuric acid, and the absorbance was recorded at 490 nm. The results were quantified based on the standard curve, prepared from the oxidized and reduced albumin.

#### Plasma protein thiol groups

The level of protein thiol groups in plasma was estimated colorimetrically, using 5,5′-dithiobis(2-nitro-benzoic acid – DTNB (the Ellman’s reagent) and quantified based on the molar extinction coefficient of 2-nitro-5-thiobenzoic ions (TNB^2−^), yellow-colored products of reaction between the DTNB and protein sulfhydryl groups (1.36 × 10^4^ M^− 1^ × cm^− 1^) [28]. 

#### Lipid peroxidation

Lipid peroxidation level in plasma was estimated based on the lipid hydroperoxides and the levels of thiobarbituric acid-reactive substances (TBARS), providing data on intermediate and end products of the lipoperoxidation reactions, respectively. Lipid hydroperoxides were detected using the ferric-xylenol orange hydroperoxide assay, described by Gay and Gebicki [29] and expressed as the hydrogen peroxide equivalents. TBARS concentration was determined using the molar extinction coefficient of malondialdehyde (1.56 × 10^5^ M^− 1^ × cm^− 1^) [30]. 

#### Total 3-nitrotyrosine in plasma proteins

The total pool of 3-nitrotyrosine in plasma proteins was estimated with the competitive ELISA, according to our previously described and optimized protocol [31]. Results were quantified and expressed as equivalents of the 3-nitrotyrosine-containing protein standard per mg of plasma protein (i.e., nanomoles of 3NT-FG/mg of plasma protein).

#### Nitric oxide and nitrate/nitrite assay

Measurements were carried out using a colorimetric reagent kit, i.e., Parameter Total Nitric Oxide and Nitrate/Nitrite Assay (# KGE001, R&D Systems^®^, Inc., Minneapolis, MN, USA), applicable for the quantitative determination of nitric oxide concentrations in plasma. The assay enables detection of nitric oxide, based on the nitrate reductase-catalyzed conversion of nitrate to nitrite. The reaction is followed by colorimetric measurement (λ = 540 nm) of the nitrite concentration, an azo dye product of the Griess Reaction. Measurements were executed following the protocol provided by the manufacturer.

### Fibrinogen modifications

#### Immunodetection of the 3-nitrotyrosine-containing fibrinogen

The 3-nitrotyrosine-containing fibrinogen was detected by the sandwich ELISA. In the first day of the assay, 96-well plates (high binding) were coated with 50 µl of anti-3-nitrotyrosine antibody solution (1:10 000; in 50 mM carbonate buffer, pH 9.6) and incubated for overnight at 4 °C. The next day, the antibody solution was removed, and the well surface was blocked with defatted milk solution (3%, in 0.05 M tris-buffered saline, enriched with 0.1% Tween 20 - TBST; 200 µl/well) for 2 h, at 37 °C. After the milk solution was removed, microplate wells were washed three times with 0.05 M TBST (200 µl/well). Then, 50 µl of the samples were layered into microplate wells and incubated for overnight at 4 °C. Following the incubation, microplates were washed three times with 0.05 M TBST (200 µl/well). Then, 100 µl of the anti-fibrinogen antibody (1: 60 000, in 0.05 TBST-based 3% milk solution), conjugated with the horseradish peroxidase was added, and the samples were incubated at 37 °C for 2 h. After the incubation, unbound antibodies were removed by washing 3-times with 0.05 M TBST (200 µl/well). Then, 100 µl/well of the SIGMAFAST™ OPD (*o*-phenylenediamine dihydrochloride) solution was added to visualize the reaction. The assay was stopped after 10-min incubation on the benchtop, using 50 µl of 40% sulphuric acid, and the absorbance was recorded at 490 nm. The standard curve was prepared analogously, using the nitrated fibrinogen standard (0.1–100 nM).

#### ELISA-based immunodetection of the acetyl-lysine-containing fibrinogen

96-well microplates (high binding) were coated with 100 µl of anti-acetyl-lysine antibody solution in 0.05 M TBS, pH 9.0 (1:5 000) and incubated for overnight at 4 °C. The next day, the antibody solution was removed, and the well surface was blocked with defatted milk solution (5%, in 0.05 M TBS 200 µl/well) for 2 h, at 37 °C. Then, the milk solution was removed, and microplate wells were washed three times with 0.05 M TBST (200 µl/well). After the washing, 100 µl of the samples were layered into microplate wells and incubated overnight at 4 °C. Next, microplates were washed three times with 0.05 M TBST (200 µl/well), and 100 µl/well of the anti-fibrinogen antibody (1: 10 000, in 0.05 TBS-based 1% milk solution), conjugated with the horseradish peroxidase, was added. The microplates were incubated at 37 °C for 2 h. Then, unbound antibody was removed, and microplates were washed three times with 0.05 M TBST (200 µl/well). The reaction was visualized using the SIGMA FAST™ OPD (*o*-phenylenediamine dihydrochloride) solution (100 µl/well), and stopped after 30 min (on the benchtop) with 50 µl of 40% sulphuric acid. The absorbance was recorded at 490 nm.

### Measurements of pro-inflammatory cytokine release

Tumor necrosis factor (TNF) and interleukin 6 (IL-6) in plasma levels were measured using the sandwich-type ELISAs. For quantification of both biomarkers, the Quantikine kits for human cytokines (i.e., IL-6 - Catalog #: D6050B, TNF-α - Catalog #: DTA00D, respectively; R&D Systems Inc., Minneapolis, MN, USA) were used.

### ROTEM

Activated rotational whole blood thromboelastometry was conducted using a computerized ROTEM device (Rotation Thromboelastometry, Pentapharm GmbH, Munich, Germany, software version 1.5.3). Four ROTEM tests, i.e. INTEM, EXTEM, FIBTEM and APTEM, were performed according to the manufacturer’s instructions. The following parameters were assessed: coagulation time (CT), clot formation time (CFT), α-angle, maximum clot firmness (MCF), maximum lysis (ML), and clot lysis index at 30, 45 and 60 min (LI 30, LI 45, LI 60 respectively).

Each ROTEM test addresses the clotting process from a different angle e.g. INTEM using ellagic acid and phospholipids gives information comparable to APTT, EXTEM using tissue factor activator provides information similar to that of PT. The ROTEM parameters reflect phases of the coagulation process by indicating clot initiation (CT), amplification (CFT and alpha angle), propagation phase (MCF), clot stabilization and fibrinolysis (e.g. LI30, ML). Full details of the ROTEM laboratory technique have been provided in previous publications [32–34].

### Statistical analysis

The Mann-Whitney U-test and *Student’s t*-*test* were used to assess the significance of differences between the studied groups. The logistic regression was also used. Correlations between variables were assessed by the Spearman rank correlation coefficient (r). In all measurements, *p* < 0.05 was considered statistically significant. Analyses were performed using STATISTICA v. 13.1 software (StatSoft, Tulsa, OK, USA).

## Results

### Assesment of various oxidative stress biomarkers

**Fig. 1:**
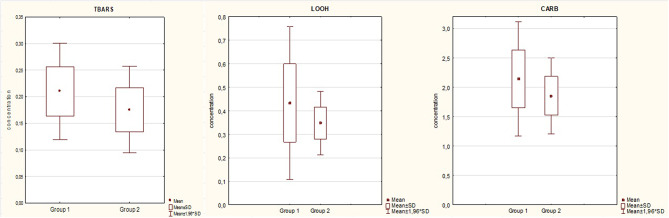
Various oxidative stress biomarkers concentrations comparison between Group 1 and 2. TBARS- thiobatrbituric acid-reactive substances [nM]; LOOH – lipid hydroperoxides [µM], CARB-Carbonyl groups [nmol C = O/mg protein]. Mean, standard deviation

In Group 1 thiobatrbituric acid-reactive substances (TBARS) concentrations were found to be significantly higher than in Group 2 (*p* = 0,002). Also levels of lipid hydroperoxides (LOOH) were markedly higher than in Group 2 (*p* = 0,035). Carbonyl group levels were significantly higher in Group 1 than in Group 2 (*p* = 0,018) (Fig. 1; Table 2). All these parameters were significant in logistic regression (Tables 3, 4 and 5). TBARS and LOOH differences remained significant regardless of the age and gender of the participants in multivariate logistic regression analysis (Table 6).

**Table 2:** Various oxidative stress biomarkers concentrations comparison between group 1 and 2

	Mean levels Group 1	Mean Levels Group 2	Median Group 1	Median Group 2	Standard deviation Group 1	Standard deviation Group 2	*P*
TBARS	0,21	0,18	0,20	0,17	0,042	0,041	0,002170
LOOH	0,43	0,35	0,38	0,35	0,167	0,068	0,035836
CARB	2,14	1,85	2,14	1,75	0,493	0,329	0,018437

TBARS- thiobatrbituric acid-reactive substances [nM]; LOOH - lipid hydroperoxides [µM], CARB-Carbonyl groups [nmol C = O/mg protein]. Mean, median, standard deviation, p-statistical significance

**Table 3:** Logistic regression carbonyl groups

	Estimate	Standard error	95% confidence limits		Wald Chi-square	OD	OD	*p*-value
CARB	-1,592	0,707	-3,011	-0,1744	5,073	0,002	0,70	0,024
Chi-square = 5,830883 df = 1 *p* = 0,016								

CARB-Carbonyl groups, OD- odds ratio, p-statistical significance

**Table 4:** Logistic regression TBARS

	Estimate	Standard error	95% confidence limits		Wald Chi-square	OD	OD	*p*-value
TBARS	-22,200	8,045	-38,34	-6,06	7,614	0,002	0,269	0,0057
Chi-square = 9,961411 df = 1 *p* = 0,0016								

TBARS- thiobatrbituric acid-reactive substances, OD- odds ratio, p-statistical significance

**Table 5:** Logistic regression LOOH

	Estimate	Standard error	95% confidence limits		Wald Chi-square	OD	OD	*p*-value
LOOH	-5,982	2,868	-11,73	-0,230	4,35	0,000697	0,867	0,04
Chi-square = 6,033576 df = 1 *p* = 0,014								

LOOH - lipid hydroperoxides OD- odds ratio, p-statistical significance

**Table 6:** Logistic regression – TBARS, CARB, LOOH, age, gender

	Estimate	Standard error	Wald Chi-square	95% confidence limits		OR	*p*-value
TBARS	-24,42	9,1332	7,15	2,6467E-19	0,0023	0,247 E-9	0,0075
CARB	-1,329	0,85	2,44	0,0479	1,46	0,265	0,118
LOOH	-8,669	3,857	5,05	0,0739E-6	0,399	0,00017	0,025
Age	-0,0227	0,0247	0,845	0,93	1,027	0,9775	0,36
Gender	-1,04	1,104	7,15	0,038	3,226	0,35	0,34
Chi-square = 22,37952 df = 5 p =,0004447							

TBARS- thiobatrbituric acid-reactive substances; LOOH - lipid hydroperoxides, CARB-Carbonyl groups; OD- odds ratio, *p*-value- statistica significance

**Fig. 2:**
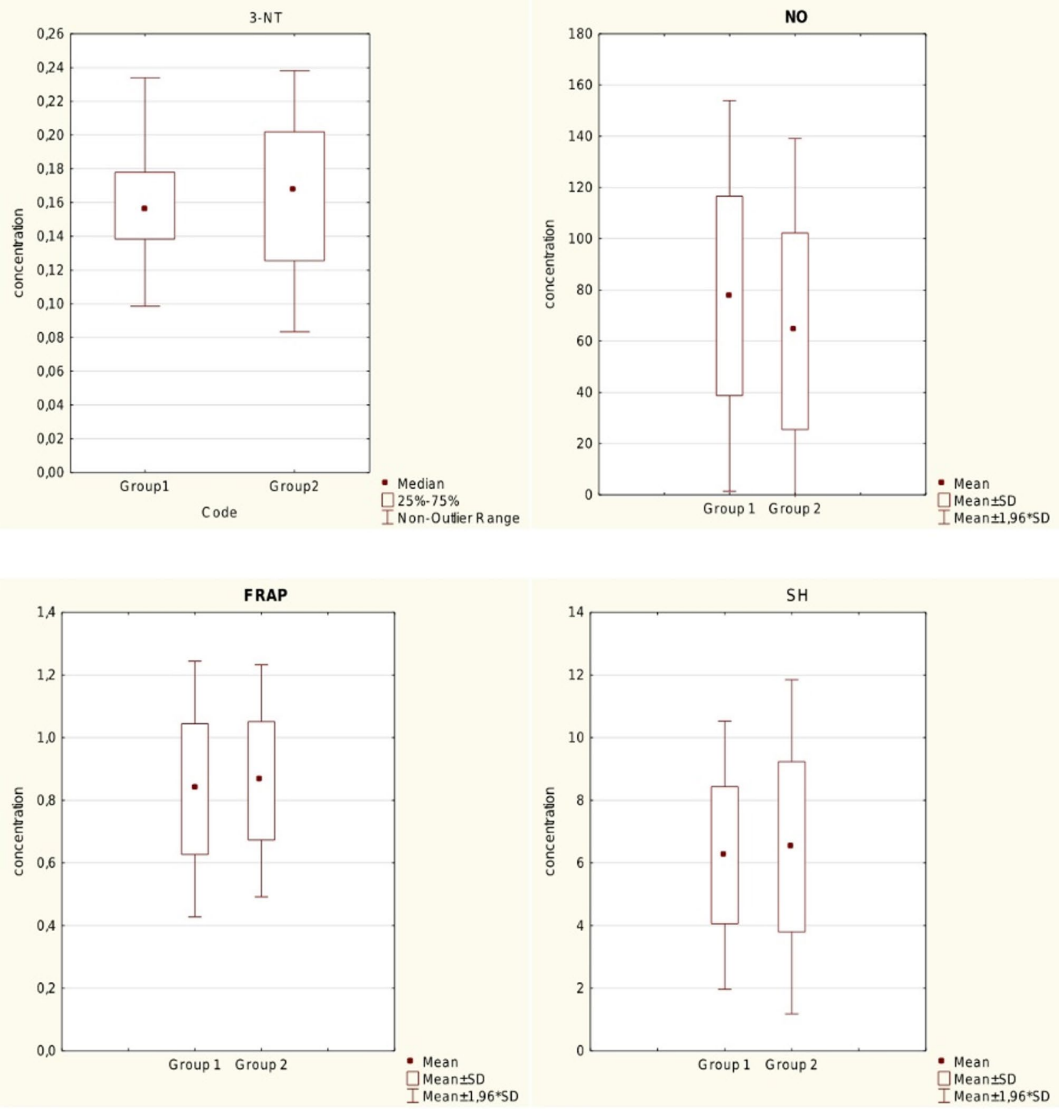
Other oxidative stress biomarkers concentration- comparison between Group 1 and 2: 3-NT-total 3-nitrotyrosine content in plasma proteins [nM], NO-nitrate concentration [µmol/ml], FRAP- ferric reducing ability of plasma [mM Fe^2+^], SH- sulfhydryl residues [µmol/ml]. Mean, standard deviation

However, other oxidative stress biomarkers such as SH groups in plasma proteins, total and 3-nitrotyrosine content in plasma proteins did not differ markedly between the studied groups (Fig. 2; Table 7). Quantitative determination of NO level in plasma indicated slightly, but not significantly higher values of the NO level in Group 1 than in Group 2. To assess the non-enzymatic capacity of plasma, the ferric reducing ability assay was used. No significant differences in this parameter between the two groups were observed.

**Table 7:** Other oxidative stress biomarkers concentration- comparison between group 1 and 2

	Mean levels Group 1	Mean Levels Group 2	Mediana Group 1	Mediana Group 2	Standard deviation Group 1	Standard deviation Group 2	*p*
3-NT	0,162037	0,162820	0,163	0,165	0,03	0,04	ns
NO	77,696	63,898	65,07	46,27	38,92	38,37	ns
SH	6,25	6,52	6,16	5,68	2,19	2,72	ns
FRAP	0,836	0,862	0,85	0,90	0,2	0,19	ns

3-NT-total 3-nitrotyrosine content in plasma proteins [nM], NO-nitrate concentration [µmol/ml], FRAP- ferric reducing ability of plasma [mM Fe^2+^], SH- sulfhydryl residues [µmol/ml]. Mean, standard deviation; p-statistical significance; ns-non significant

Table 8 shows the results of stress activity parameters arranged according to the amount of detected antibodies (single vs. triple) in Group 1. ”1 APS antibody” means that in 23 patients from Group 1 only single antibody was positive (LAC or aβ2GPI or aCL) and ”3 APS antibodies” means that in 6 patients from Group 1 all assessed antibodies were positive (LAC + aβ2GPI + aCL). Intensity of oxidative stress seems to depend on the number of APS antibodies (single or triple positive APS), however it was not statistically significant.

**Table 8:** Concentrations of oxidative stress and number of 1 and 3 APS antibodies (single or triple positive)

	TBARS [nM]	LOOH [µM]	NO [µmol/ml]	TNF [pg/ml]	CARB [nmol C = O/mg protein]	3-NT-FB [nM]	Acetyl-LYS-FB [OD units]
1 APS antibody (*n* = 23)	0.219	0.403	83.14	27.42	2.11	45.14	0.26
3 APS antibodies (*n* = 6)	0.214	0.507	85.50	34.86	2.24	70.88	0.29

TBARS - thiobarbituric acid-reactive substances [nM]; LOOH - lipid hydroperoxides [mM], Carbonyl groups [nmol C = O/mg protein], 3-NT-FB − 3-nitrotyrosine-containing fibrinogen [nM], NO - nitric oxide concentration [µmol/ml], TNF - tumor necrosis factor [pg/ml]

### Fibrinogen modifications

**Fig. 3:**
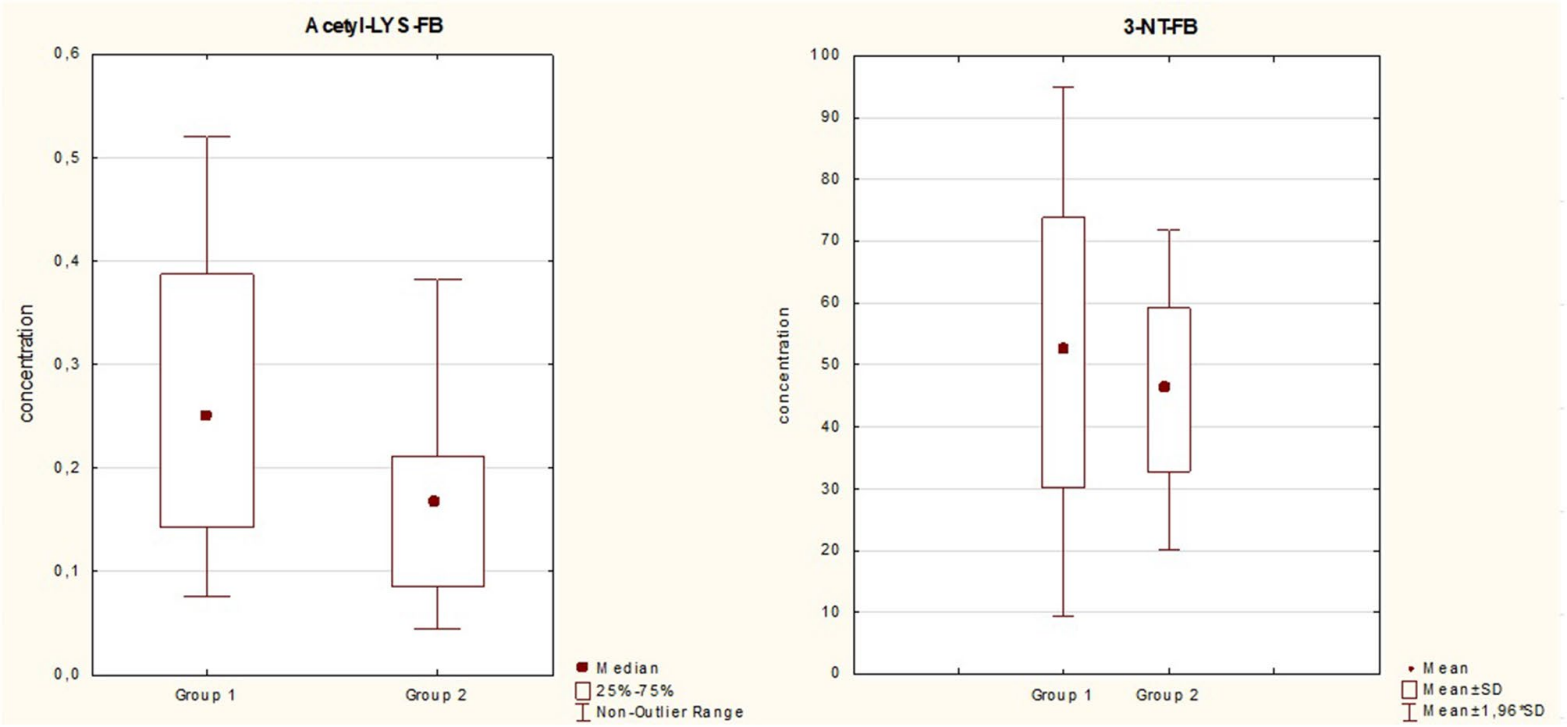
Fibrinogen modifications: measurements of the acetyl-lysine-containing fibrinogen (acetyl-LYS-FB; OD units] and the 3-nitrotyrosine-containing fibrinogen in plasma (3-NT-FB; nM). Mean and standard deviation

The levels of the acetyl-lysine-containing fibrinogen (acetyl-LYS-FB) were significantly higher in Group 1 than in Group 2 (*p* = 0,0028) (Fig. 3; Table 9). The differences between the groups in terms of 3-nitrotyrosine content in plasma proteins and fibrinogen itself were not significant. There was a slight tendency towards a higher concentration of 3-nitrotyrosine groups in fibrinogen was observed in Group 1, however it was not statistically significant.

**Table 9:** Fibrinogen modifications-comparison between group 1 and 2

	Mean levels Group 1	Mean Levels Group 2	Mediana Group 1	Mediana Group 2	Standard Deviation Group 1	Standard deviation Group 2	*p*
acetyl-LYS-FB	0,26	0,17	0,25	0,17	0,14	0,09	0,0028
3-NT-FB	52,07	45,99	44,64	49,55	31,83	13,20	ns

acetyl-LYS-FB- acetyl-lysine-containing fibrinogen (OD units] and 3-NT-FB- the 3-nitrotyrosine-containing fibrinogen in plasma (nM). p-statistical significance, ns- non significant

Fibrinogen modification markers were also correlated with the number of positive antibodies in Table 8, however no significant differences were found.

### Inflammation markers

**Fig. 4:**
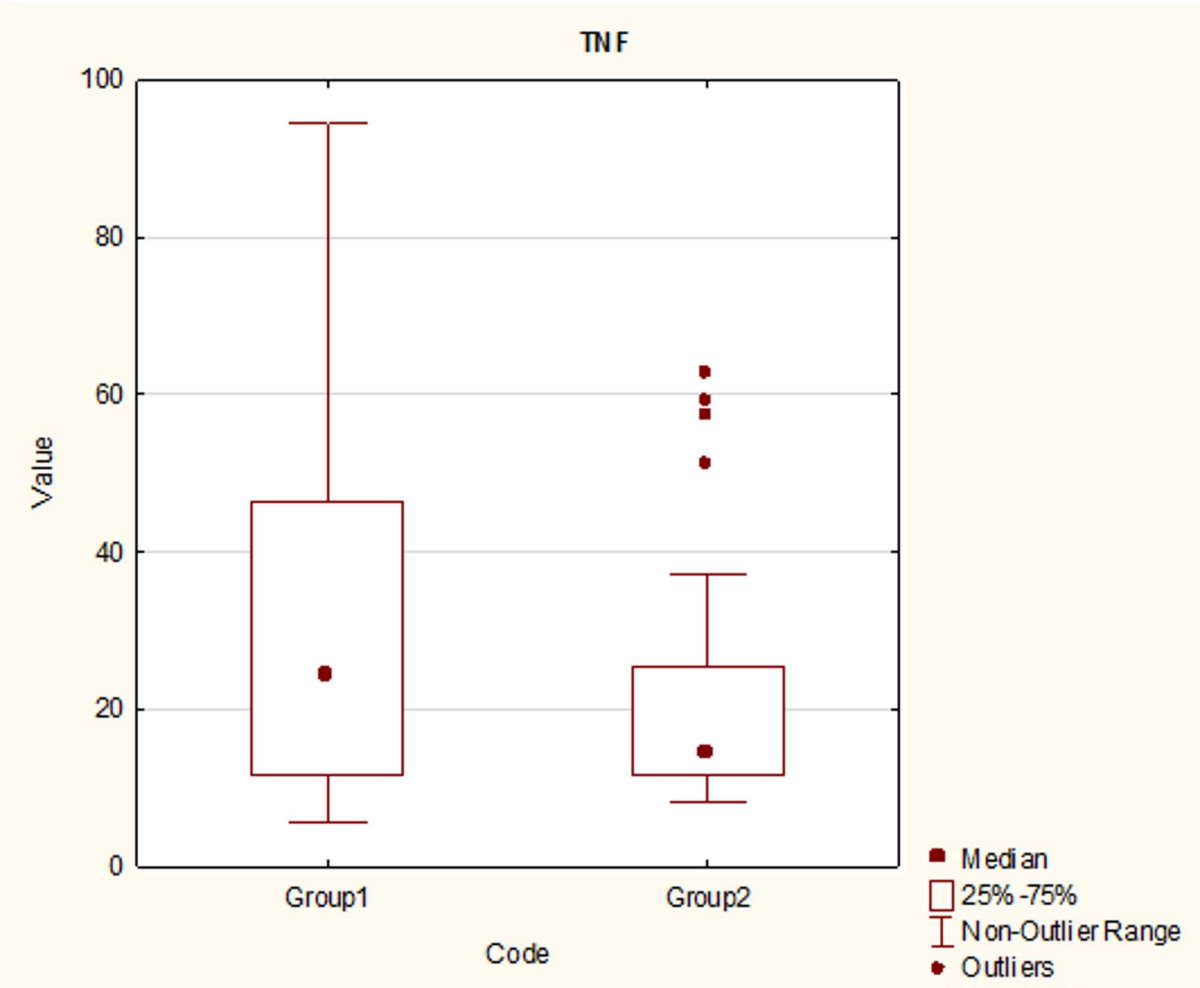
TNF-tumor necrosis factor (pg/ml). Mean and standard deviation

Levels of C-reactive protein and IL-6 were not significantly different between groups. Levels of tumor necrosis factor were markedly higher in Group 1 than in Group 2, albeit this difference was not statistically significant in univariate logistic regression (Fig. 4.)

### ROTEM parameters

CT readings were found to be markedly higher in Group 1 than in Group 2 according to INTEM, EXTEM, APTEM and FIBTEM. Concurrently, CFT was shorter in Group 1 than in Group 2 according to the APTEM and EXTEM tests. Alpha angle value was also markedly lower in Group 1 than in Group 2 in the INTEM test (77 vs. 79). No marked differences were found between the Groups regarding MCF readings and lysis parameters. The ROTEM values are displayed in Tables 10 and 11. There were no significant correlation between ROTEM parameters and level of oxidative and nitrosative stress biomarkers.

**Table 10:** ROTEM results. Data expressed as median, range

ROTEM test		CT [s]	*P*	CFT [s]	*P*	Alpha (°)	*P*
INTEM	Group 1	212 (122–273)	0.00	65 (34–86)	ns	77 (72–83)	0.04
	Group 2	171 (147–205)		58 (42–77)		79 (75–81)	
EXTEM	Group 1	72.5 (50–132)	0.00	63.5 (51–77)	0.01	74.5 (63–83)	ns
	Group 2	61 (42–88)		110 (67–128)		71 (65–81)	
APTEM	Group 1	72 (28–109)	0.00	92 (44–124)	0.02	74.5 (67–83)	ns
	Group 2	62 (50–81)		100 (66–134)		74 (66–80)	
FIBTEM	Group 1	68 (49–118)	0.00	1620 (54-9946)	ns	72 (27–83)	ns
	Group 2	58 (48–76)		651.5 (232–1071)		71 (56–79)	

CT - coagulation time; CFT - clot formation time, α-angle in Group 1 and in Group 2; ns - not significant

**Table 11:** ROTEM results. Data expressed as median, range

ROTEM test		MCF (mm)	*p*	LI 45[%]	*p*	LI60 [%]	*P*	ML (%)	*p*
INTEM	Group 1	66 (58–76)	ns	96 (92–100)	ns	93 (87–98)	ns	17,5 (8–25)	ns
	Group 2	66 (60–76)	ns	96 (91–99)		93 (86–97)		17 (11–22)	
EXTEM	Group 1	64 (51–77)	ns	97 (94–99)	ns	93 (89–97)	ns	19 (10–28)	ns
	Group 2	63 (54–74)	ns	97 (90–99)		92 (86–96)		21 (13–25)	
APTEM	Group 1	64 (57–78)	ns	97 (93–99)	ns	93,5 (88–96)	ns	18,5 (5–28)	ns
	Group 2	64 (56–75)	ns	96 (90–99)		92 (87–97)		17 (8–25)	
FIBTEM	Group1	15 (8–35)	ns	100 (90–100)	ns	100 (97–100)	ns	0 (0–25)	ns
	Group 2	14 (8–26)	ns	100 (94–100)		100 (98–100)		0 (0–13)	

MCF - maximum clot firmness; ML - maximum lysis; LI 45 - clot lysis index at 45 min.; LI 60 - clot lysis index at 60 min. in Group 1 and in Group 2; ns - not significant

### Clinical follow -up

After blood collection, the participants were followed for two years in the outpatient clinic. In Group 2 there was no thromboembolic event. In Group 1 two participants experienced thrombotic complications, the first one had transient ischemic attack (before the incident he was positive in LAC and aCL antibodies), the other one had pulmonary embolism (before was single positive in LAC). Both patients after the thrombotic event are treated with anticoagulants.

## Discussion

The literature evidence indicates that not only oxidative, but also nitrative and nitrosative modifications of different biomolecules may contribute to different abnormalities occurring in immune diseases [35, 36]. Enhancement of nitration and/or nitrosylation of biomolecules may impair their physiological functions, modulate cell signaling pathways, and even lead to the loss of immune tolerance and development of autoimmunity [37]. Nitrosative stress molecules have been suggested to be potential diagnostic biomarkers in some diseases with immune etiology [38]; however none of oxidative stress biomarkers, or their panel, have been definitively matched to the pathology of APS so far.

Our study demonstrated that the levels of certain oxidative stress biomarkers such as TBARS, LOOH and carbonyl groups were markedly higher in the group of patients in whom the presence of antiphospholipid antibodies was confirmed compared to the group of healthy volunteers.

Previous studies also reported increased lipid peroxidation biomarkers [14, 39, 16] and decreased paraoxonase 1 activity (PON1) [18]. PON1 is a hydrolytic enzyme which prevents lipid oxidation. Although those studies assessed different oxidative biomarkers, our results are corresponding. Stanisavljevic et al. [17] proved that lipid peroxidation could be an independent predictor for endothelial dysfunction in APS patients. One of the assessed markers in Stanisavljevic’s study was LOOH, the same parameter as in our study, and it was also found to be increased. Interestingly, Sciascia et al. [15] found significant correlation between the increased stress activity and the amount of antibodies (single vs. triple). In our study the levels of stress biomarkers seem to have depended on the number of antibodies (single- vs. triple-positive APS antibodies detected), however the individual study subgroups in relation to the levels of APS antibodies were too small to detect significance.

In our study the quantitative determination of NO level in plasma did not significantly differ between the studied groups. Previous studies [40] described impaired synthesis of nitric oxide (NO). NO may act as an inhibitor of LDL oxidation, which can also protect endothelial cells against the toxic effects of oxidized LDL (oxLDL) [41].

One of the best known markers of nitrative stress in people is 3-nitrotyrosine, which is also considered a fingerprint of formation and action of peroxynitrite, a reactive oxygen (and nitrogen) species. Since the formation of peroxynitrite requires a simultaneous generation of nitric oxide and superoxide anion [42], this molecule is a bridge linking two types of biochemical stress, i.e. oxidative and nitrative stress. According to the literature data, in animals injected with aCL antibodies, an increase in serum 3-nitrotyrosine (considered a marker of nitrative stress) was observed. Furthermore, experiments on endothelial cells, conducted to elucidate the molecular mechanisms linking APS markers with the occurrence of nitrative stress, revealed that exposure of these cells to aCL antibodies induced the expression of iNOS, and in consequence increased the generation of nitric oxide [43]. Moreover, in other studies employing the cellular model, Sacharidou [44] showed that aPLs inhibit the endothelial isoform of NOS (eNOS) activation, significantly decreasing the modulatory functions of the endothelium. Among the three types of the NOS enzyme (i.e., neuronal NOS, inducible NOS, and endothelial NOS), only the eNOS is a modulator of vascular functions and exerts anti-atherogenic and anti-thrombotic effects by preventing monocyte/leukocyte interactions with endothelium and platelet activation.

Since in the previous studies the NO levels were reduced and were not a good marker of nitrative stress, we decided to assess the levels of nitrotyrosine and fibrinogen containing 3-nitrotyrosine. Fibrinogen is extremely susceptible to modifications by reactive oxygen species or nitrogen species, making it a sensitive marker of such changes [45, 46]. 3-nitrotyrosine-containing fibrinogen was detected in patients with either severe or chronic inflammation [47]. Its presence was also considered as a potential biomarker of oxidative stress in venous thromboembolism [48]. Nitrotyrosine levels were increased in APS patients [40, 49]. In our study, the levels of total 3-nitrotyrosine content in plasma proteins in both groups were similar. The concentration of nitrated fibrinogen in the group of patients with phospholipid antibodies was higher, but the difference was not significant.

Oxidative stress may also cause conformational changes in protein by promoting postranslational modifications (PTMs), which in APS mainly involve β2GPI [4]. β2GPI is a plasma glycoprotein, which plays an important role in the blood coagulation system [50]: inhibits the FXII-dependent activation of fibrinolysis, inhibits the inactivation of the activated factor V, inhibits the interaction between platelets and VWF leading to a decrease in platelet adhesion, binds to t-PA acting as a cofactor for the generation of plasmin. β2GPI is also a major antigen for autoantibodies involved in antiphospholipid syndrome. In normal conditions 99% of plasma β2GPI is in closed conformation [51], which prevents antibodies from binding to hidden epitopes located in an inaccessible part of this molecule. However, PTMs such as acetylation, can modify structure and stability of β2GPI leading to a conformational shift to open configuration and to exposure of previously hidden epitopes. Lysine acetylation is one of the most common PTMs in proteins [52], whilst β2GP has high content of lysine residues [53]. Buchholz et al. [53] showed that closed conformation of β2GPI molecule is stabilized by the electrostatic interaction between lysine residues and that lysine acetylation shifts the equilibrium between closed and open forms of β2GPI. In our study we assessed acetylation of lysine residues fibrinogen, the most susceptible to PTMs plasma protein. The acetyl-lysine-containing fibrinogen was significantly higher in the group of people with antiphospholipid antibodies, which may indirectly suggest increased acetylation of lysine groups also in other proteins.

The obtained results of ROTEM were inconsistent. On the one hand, elongation of the initiation coagulation phase (CT) was observed in all assessed tests in the group with positive antiphospholipid antibodies, perhaps due to the interference of antibodies. On the other hand, the clot formation time (CFT) was significantly shorter (EXTEM, APTEM) in this group, as in the hypercoagulable state, however alpha angle was not increased. Also no marked differences were found in the MCF reading, so we can suppose that the quality of clot did not differ between the studied groups. Nonetheless, previous studies demonstrated that clots in APS are less permeable, thinner and with more branched fibrin fibers and therefore less susceptible to lysis [50]. Probably antiphospholipid antibodies alone are not sufficient to initiate coagulation cascade or even can interfere with the clotting, however once it starts, their presence can amplify the coagulation process resulting in excessive thrombosis. The recent studies suggested that aPLs are necessary but alone not sufficient to induce thrombosis in APS [25, 54].

Pappa et al. [55] examined the oxidative stress and DNA damage (single and double-stand breaks) in peripherial blood mononuclear cells from three groups: PAPS, asymptomatic antiphospholipid antibody (aPL) positive individuals without APS and healthy donors. Asymptomatic subjects had higher DNA damage levels and higher levels of oxidative stress (as indicated by the reduction of glutathione (GSH) to oxidized glutathione (GSSG) ratio) than healthy controls, but lower than APS patients.

In conclusion, although our study failed to demonstrate the hypercoagulable state in ROTEM, it still may be regarded of interest as we demonstrated that the levels of selected oxidative stress biomarkers, especially lipid oxidative stress biomarkers, are significantly higher in participants with antiphospholipid antibodies, with no previous thrombotic event. Although we used stress biomarkers different from those in other studies, our study also showed increased lipid peroxidation, which is probably related to reduced PON1 activity. Although we were unable to proved increased nitrotyrosine levels or even nitrated fibrinogen levels, we demonstrated increased acetylation of lysine groups in fibrinogen. Probably, as in Papa’s study [55], in the group of patients with aPLs and without thrombosis the pathological processes are not as advanced as in people with APS. However, this study confirms that oxidative stress plays an important role in the pathogenesis of APS and is not secondary to thrombosis. Even if aPLs are not sufficient alone to initiate thrombosis, they may lead to a prothrombotic state.

Even though there is strong evidence of higher thrombotic risk in APS patients, there is no consensus about how to prevent thrombotic events in such patients and how to properly estimate this risk. In a prospective controlled trial [56] low-dose aspirin proved to be no better than placebo in preventing first thrombotic event in APS patient. At the same time treatment with anticoagulants poses a higher risk of bleeding which in some patients outweighs the thrombotic risk. Better understanding of the underlying processes allows for better risk stratification and better targeted prevention, for example aimed at reducing oxidative stress.

## Conclusion

Study confirms increased levels of oxidative biomarkers and higher concentration of post-translational modifications parameter - acetyl-lysine-containing fibrinogen in patients in whom the presence of antiphospholipid antibodies was confirmed, but who had never experienced a thrombosis event.

## Data Availability

I have all data sets if it necessary, however I do not shared openly to protect study participant privacy.
